# Transcriptomic Insights into the Degree of Polymerization-Dependent Bioactivity of Xylo-Oligosaccharides

**DOI:** 10.3390/plants14192958

**Published:** 2025-09-24

**Authors:** Hanbo Wang, Tieqiang Wang, Jiakun Zhang, Lijuan Wang, Weidong Li, Zhen Wang, Jiusheng Li

**Affiliations:** 1 Hebei Technology Innovation Center of Agricultural Water Saving, Hebei Institute of Water Science, Shijiazhuang 050051, China; wanghanbo0014@163.com; 2 Biotechnology and Food Science Research Institute, Hebei Academy of Agriculture and Forestry Sciences, Shijiazhuang 050050, China; zhangjiakun0521@126.com; 3 Department of Irrigation and Drainage, China Institute of Water Resources and Hydropower Research, Beijing 100048, China; 17835423217@163.com (L.W.); wangzhen686@iwhr.com (Z.W.); lijs@iwhr.com (J.L.); 4 Hebei Yellow River Diversion Project Affairs Center, Shijiazhuang 050035, China; hbswliweidong@163.com

**Keywords:** xylo-oligosaccharide, degree of polymerization, immune response, growth, transcriptome sequencing

## Abstract

Plant cell wall-derived oligosaccharides, such as xylo-oligosaccharides (XOS), serve as key signaling molecules regulating plant growth and immunity. The bioactivity of XOS is closely tied to their degree of polymerization (DP), yet the molecular mechanisms underlying DP-specific effects remain poorly understood. Here, we investigated the transcriptional and phenotypic responses of lettuce (*Lactuca sativa*) to foliar application of four high-purity XOS variants: xylobiose (XOSY, DP2), xylotriose (XOSB, DP3), xylotetraose (XOSD, DP4), and xylopentose (XOSW, DP5). Phenotypic analyses revealed that high-DP XOS (XOSD and XOSW) significantly enhanced aboveground biomass and root system development, with XOSD showing the most pronounced effects, including a 31.74% increase in leaf area and a 20.71% increase in aboveground biomass. Transcriptomic profiling identified extensive transcriptional reprogramming across treatments, with XOSD eliciting the highest number of differentially expressed genes (DEGs). Functional enrichment analyses indicated that XOSD and XOSW upregulated genes involved in plant hormone signaling, starch and sucrose metabolism, and cell wall biosynthesis, while downregulating photosynthesis-related genes. Notably, MapMan and KEGG pathway analyses revealed that XOSD significantly activated biotic stress-related pathways, including MAPK signaling, β-1,3-glucanase activity, and PR protein pathways. In contrast, XOSY treatment primarily upregulated genes linked to basal immunity, highlighting distinct mechanisms employed by low- and high-DP XOS. These findings demonstrate that XOS with varying DP differentially modulate growth- and immunity-related processes in lettuce. High-DP XOS, particularly XOSD, not only promote plant biomass accumulation but also enhance immune responses, highlighting their potential as biostimulants for sustainable agriculture. This study provides a molecular framework for understanding the DP-specific bioactivity of XOS and their dual role in optimizing plant growth and defense.

## 1. Introduction

Cellulose and hemicellulose are the predominant polysaccharides in plant cell walls, playing crucial roles in maintaining cellular integrity and enabling adaptive responses to a wide array of biotic and abiotic stressors [1,2,3]. Upon exposure to stressors, enzymatic degradation of these polysaccharides releases oligosaccharides, such as cello-oligosaccharides (COOS) and xylo-oligosaccharides (XOS), which have emerged as key signaling molecules in plant defense and growth regulation [4]. Acting as damage-associated molecular patterns (DAMPs), these oligosaccharides are recognized by plant pattern recognition receptors, triggering immune signaling pathways and influencing fundamental physiological processes [5,6,7]. Understanding the molecular mechanisms underlying these processes is essential for advancing strategies to enhance plant resilience and improve crop productivity under stress conditions.

The bioactivity of oligosaccharides is intricately linked to their degree of polymerization (DP), which governs their interaction with specific receptors and dictates the downstream signaling pathways they activate [8]. During pathogen attack, cell wall-degrading enzymes (CWDEs) hydrolyze polysaccharides into oligomers [7,9,10,11,12]. These oligomers are recognized by plasma membrane-localized pattern recognition receptors, triggering calcium influx, mitogen-activated protein kinase (MAPK) cascades, and the transcriptional activation of immune-related genes [13,14,15,16,17,18]. Notably, DP2 COOS rapidly induce immune responses independent of reactive oxygen species (ROS), whereas higher DP variants (e.g., DP3–5) elicit more pronounced ROS production and transcriptional reprogramming, leading to enhanced disease resistance [19,20,21,22]. This DP-dependent variation in immune activation underscores the complexity of oligosaccharide-mediated signaling networks and highlights their potential for fine-tuning plant immune responses.

In addition to their roles in immunity, oligosaccharides also regulate growth-related processes by modulating key metabolic and hormonal pathways [23]. For example, COOS have been shown to influence auxin and nitrate signaling, both of which are critical regulators of biomass accumulation and resource allocation [24]. Evidence suggests that oligosaccharides with higher degrees of polymerization (DP) often exhibit stronger growth-promoting effects, indicating that DP is a key determinant in coordinating the trade-off between growth and defense [19]. Despite the extensive study of cellulose-derived oligosaccharides, the regulatory potential of hemicellulose-derived XOS remains relatively unexplored.

This study systematically investigated the degree of polymerization (DP)-specific effects of four high-purity xylo-oligosaccharide (XOS) variants—xylobiose (XOSY), xylotriose (XOSB), xylotetraose (XOSD), and xylopentose (XOSW)—on lettuce (*Lactuca sativa*) seedlings. Integrating transcriptomic profiling with phenotypic analyses, we uncovered key regulatory pathways and genes influenced by XOS treatments, including those involved in auxin signaling, nitrate transport, and immunity-related transcriptional networks. Among the variants, XOSD induced the most extensive transcriptional reprogramming and pronounced phenotypic changes, underscoring its dual role in promoting plant growth and enhancing immune responses. These findings establish a molecular framework for understanding the DP-specific bioactivity of XOS and highlight their potential as innovative biostimulants for advancing sustainable agricultural practices.

## 2. Results

### 2.1. Effects of Xylo-Oligosaccharides with Different Degrees of Polymerization on Lettuce Growth

**Figure 1:**
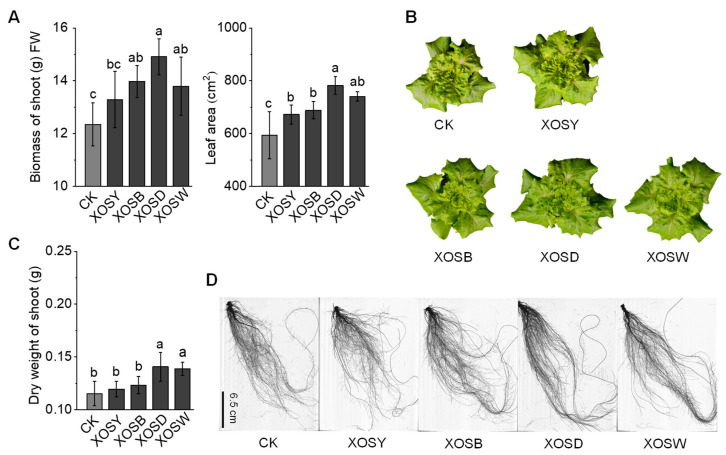
Effects of xylo-oligosaccharides with different degrees of polymerization on the growth of lettuce (27 d). (**A**) Aboveground fresh biomass and leaf area of lettuce seedlings treated with distilled water (CK), xylobiose (XOSY), xylotriose (XOSB), xylotetraose (XOSD), and xylopentose (XOSW). (**B**) Representative images of lettuce plants under CK, XOSY, XOSB, XOSD, and XOSW treatments. (**C**) Dry weight of lettuce seedlings treated with XOS. (**D**) Representative root images of lettuce seedlings showing differences in root morphology under CK, XOSY, XOSB, XOSD, and XOSW treatments. Values are presented as mean ± standard deviation (*n* = 8). Significant differences between treatments and the control group are indicated by different letters (*p* < 0.05). Scale bar for root images: 6.5 cm.

To evaluate the degree-dependent effects of xylo-oligosaccharides (XOS) on plant performance, we assessed shoot and root growth parameters in lettuce following foliar application of XOS with varying DP. High-DP XOS treatments significantly enhanced aboveground growth relative to the distilled water control (CK) (Figure 1A,B). Xylotetraose (DP4, XOSD) increased shoot fresh weight by 20.71% and expanded leaf area by 31.74%, while xylopentaose (DP5, XOSW) resulted in respective increases of 11.68% and 24.79% (*p* < 0.05). In contrast, low-DP XOS treatments, including xylobiose (DP2, XOSY) and xylotriose (DP3, XOSB), did not significantly affect biomass or leaf area compared to CK (*p* > 0.05), indicating a lack of measurable impact on shoot performance under the tested conditions.

Root morphological traits displayed a similar trend (Figure 1C,D, Table S1). DP4 treatment led to the largest enhancements, increasing total root length by 18.77%, root surface area by 19.15%, lateral root length by 38.82%, and adventitious root length by 21.08% (*p* < 0.05). DP5 also significantly improved root parameters, though to a lesser extent. By contrast, DP2 and DP3 treatments did not result in significant changes across measured root traits (*p* > 0.05).

Collectively, these results demonstrate that the bioactivity of XOS in promoting lettuce growth is highly dependent on polymer length. Among the tested compounds, xylotetraose (DP4) consistently produced the most pronounced improvements in both shoot biomass and root architecture.

### 2.2. Quality Assessment of RNA-Seq Data

To assess transcriptomic responses to XOS treatment, RNA sequencing was performed on 15 libraries derived from CK, XOSY, XOSB, XOSD, and XOSW treatments, each with three biological replicates. After quality filtering, each library yielded between 36.4 and 53.5 million clean reads, with an average Q30 score exceeding 92.8%, indicative of high base-calling accuracy (Table S2). The mean GC content was 45.65%, and all samples exhibited genome alignment rates above 92.94%, reflecting excellent sequencing quality and reference compatibility.

Biological replicates within each group displayed strong internal consistency, with Pearson correlation coefficients greater than 0.92 across all treatments (Figure S1A). Principal component analysis (PCA) revealed clear separation of treatment groups, with PC1 accounting for 64.03% of the total variance (Figure S1B). Hierarchical clustering of global expression patterns confirmed treatment-specific transcriptomic profiles and showed marked divergence between XOS-treated samples and the control group (Figure S1C). These metrics confirm that the RNA-seq dataset is of high quality and exhibits strong reproducibility. The well-resolved sample clustering further supports the existence of degree-specific transcriptomic responses to XOS application in lettuce.

### 2.3. Differentially Expressed Gene (DEG) Analysis

**Figure 2:**
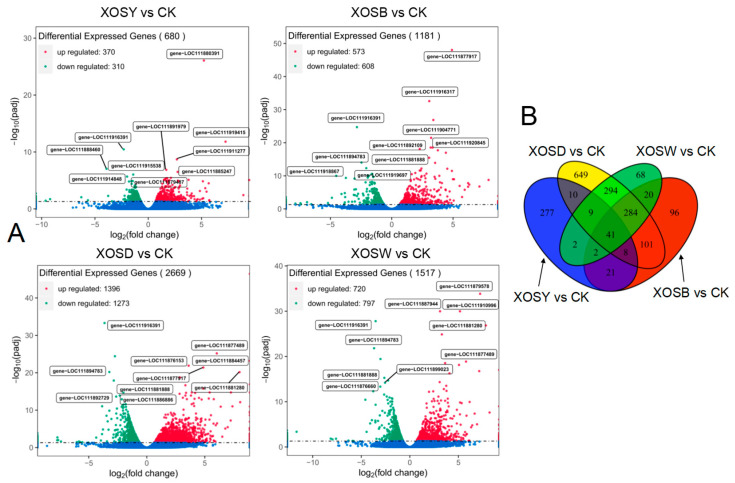
Volcano plot (**A**) and Venn diagram (**B**) analysis of differentially expressed genes (DEGs) in lettuce leaves treated with xylo-oligosaccharides of low degree of polymerization (DP 2–5). The volcano plot highlights up-regulated (red) and down-regulated (green) genes, comparing samples with and without xylo-oligosaccharide treatment. The Venn diagram illustrates the overlap and uniqueness of DEGs across different treatment conditions.

Transcriptome profiling revealed extensive and degree-dependent transcriptional reprogramming in response to XOS treatment (Figure 2). Under a false discovery rate (FDR) threshold of *q* < 0.05, the number of differentially expressed genes (DEGs) varied markedly across treatments. The XOSD group exhibited the strongest transcriptional shift, with 1396 upregulated and 1273 downregulated genes (Figure 2A). This was followed by XOSW, which induced 720 upregulated and 797 downregulated genes. XOSB yielded 573 upregulated and 608 downregulated genes, while XOSY showed the fewest DEGs (370 up, 310 down). Notably, this trend was accompanied by differential expression of key transcription factors associated with plant development and immunity. For example, MYB12, a regulator of flavonoid biosynthesis and tissue expansion, and SPEECHLESS (SPCH), a bHLH transcription factor involved in stomatal patterning, were both markedly induced under XOSD treatment, consistent with enhanced growth signaling. In contrast, WRKY40, a canonical immune-related TF, was preferentially upregulated in the XOSY group, suggesting a chain length–dependent partitioning of transcriptional programs.

A shared DEG subset analysis identified 41 genes that were differentially regulated across all four XOS treatments, suggesting the existence of a core transcriptional response to XOS exposure regardless of chain length. In contrast, treatment-specific DEGs varied widely: XOSD induced 649 unique DEGs, followed by XOSY (277), XOSB (96), and XOSW (68) (Figure 2B). These results highlight both the quantitative magnitude and the qualitative specificity of transcriptional responses to XOS with increasing polymerization degree.

### 2.4. GO Enrichment Analysis

**Figure 3:**
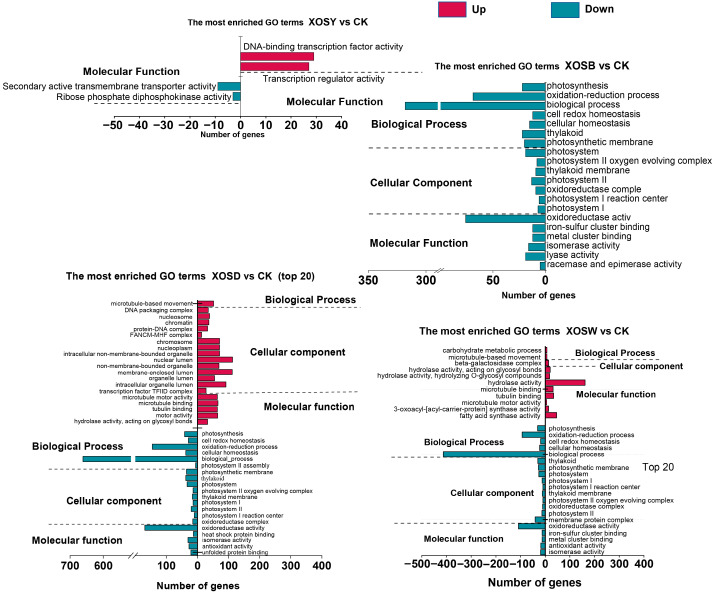
GO enrichment analysis of differentially expressed genes (DEGs) in lettuce leaves treated with xylo-oligosaccharides (DP 2–5). The analysis categorizes DEGs into enriched Gene Ontology (GO) terms across three domains: biological processes, cellular components, and molecular functions, highlighting key functional pathways affected by the treatment.

To explore the functional implications of differentially expressed genes (DEGs) under XOS treatment, Gene Ontology (GO) enrichment was conducted across three categories: biological process (BP), cellular component (CC), and molecular function (MF) (Figure 3).

XOSY induced minimal enrichment, with only four significantly enriched GO terms (FDR < 0.05), all within the molecular function category. These included terms related to DNA-binding transcription factor activity and transcriptional regulation.

XOSB treatment did not yield significant enrichment for upregulated DEGs. However, the top 20 GO terms among downregulated genes included several associated with photosynthesis and chloroplast organization. These encompassed BP terms such as photosynthesis and light harvesting, CC terms such as thylakoid membrane and photosystem I reaction center, and MF terms including oxidoreductase activity and iron-sulfur cluster binding.

XOSD elicited the most extensive GO enrichment across both up- and downregulated DEGs. Among upregulated genes, 20 GO terms were significantly enriched, including the BP term microtubule-based movement, CC terms such as nucleosome, chromatin, and non-membrane-bound organelle, and MF terms related to microtubule motor activity and hydrolase activity. For downregulated DEGs, six BP terms were enriched, including photosynthesis, redox homeostasis, and cellular metabolic process, alongside nine CC terms (e.g., photosystem II, photosynthetic membrane, and thylakoid) and five MF terms (e.g., oxidoreductase activity, heat shock protein binding, and antioxidant activity).

XOSW also induced broad enrichment. Upregulated DEGs were significantly enriched in 11 GO terms, including BP terms such as carbohydrate metabolism and microtubule-based movement. Enrichment in CC and MF categories highlighted terms such as β-galactosidase complex, hydrolase activity, and microtubule motor activity. Downregulated DEGs showed enrichment in 20 GO terms, with BP terms including redox processes and cellular homeostasis, 10 CC terms related to photosynthetic systems, and MF terms such as iron-sulfur cluster binding, oxidoreductase activity, and antioxidant activity.

Together, these data demonstrate that XOS treatments, particularly DP4 and DP5, extensively modulate gene networks associated with photosynthesis, redox homeostasis, cytoskeletal organization, and protein complex assembly. While lower-DP XOS induced minimal functional changes, higher-DP variants provoked broad transcriptional reprogramming across multiple functional domains.

### 2.5. KEGG Pathway Enrichment Analysis

**Figure 4:**
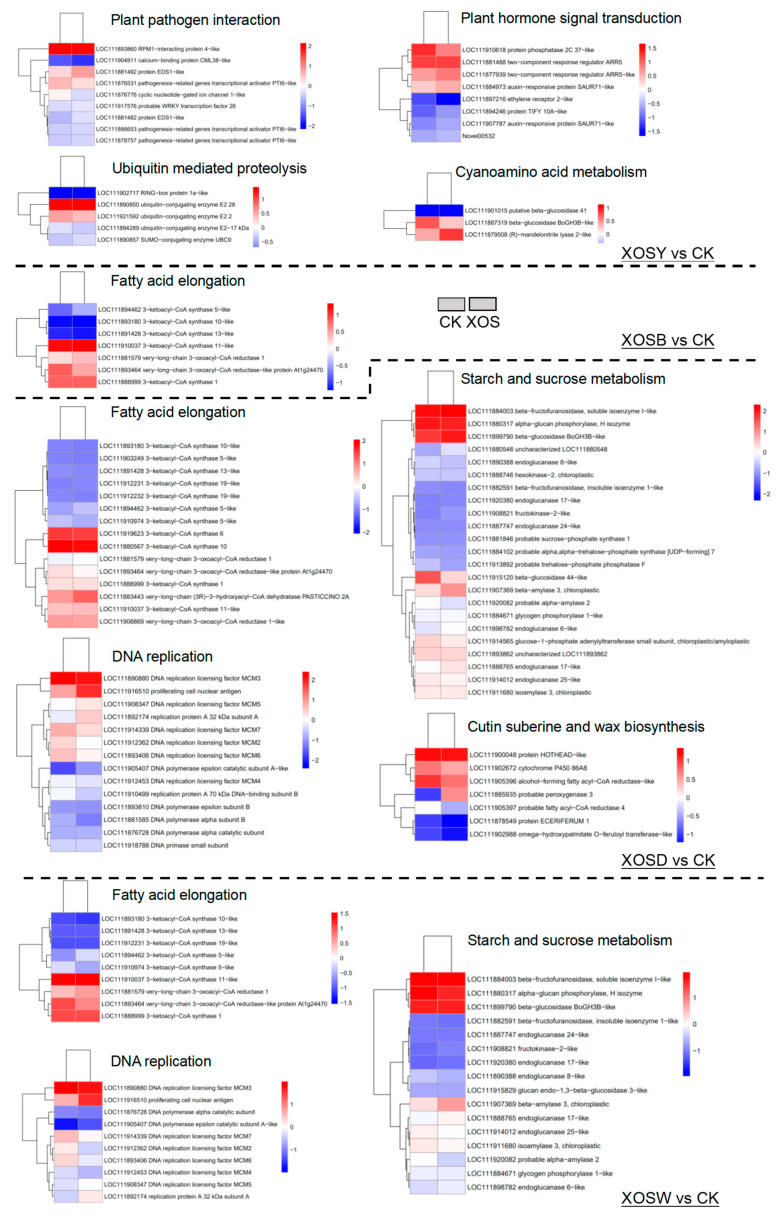
KEGG enrichment analysis of differentially expressed genes (DEGs) in lettuce leaves treated with xylooligosaccharides (DP 2–5).

To identify functional pathways regulated by XOS of varying polymer lengths, KEGG pathway enrichment analysis was conducted based on DEGs across treatments (Figure 4).

Low-DP XOS treatments (DP2 and DP3) exhibited limited but distinct enrichment patterns. For XOSY, 370 upregulated DEGs were significantly enriched in immune-related pathways, including plant–pathogen interaction, plant hormone signal transduction, and ubiquitin-mediated proteolysis. Although 310 downregulated DEGs were mapped to 76 pathways, no significant enrichment was observed. In the XOSB group, 573 upregulated DEGs were significantly enriched in fatty acid elongation, cutin, suberin, and wax biosynthesis, secondary metabolite biosynthesis, circadian rhythm, and starch and sucrose metabolism. Downregulated DEGs showed significant enrichment in photosynthesis-related pathways, including photosystem antenna proteins, carbon fixation, and porphyrin and chlorophyll metabolism.

High-DP XOS treatments (DP4 and DP5) elicited broader transcriptional reprogramming. The XOSD group displayed the most extensive enrichment, with 1396 upregulated DEGs significantly enriched in 94 pathways. Notable pathways included DNA replication, fatty acid elongation, cutin, suberin, and wax biosynthesis, and starch and sucrose metabolism. The 1273 downregulated DEGs were significantly enriched in multiple photosynthesis-related processes, such as antenna proteins, thylakoid membrane, carbon fixation, and also in pentose phosphate, porphyrin and chlorophyll metabolism, and amino acid metabolism, including glycine, serine, and threonine metabolism. Similarly, XOSW upregulated 720 genes significantly enriched in pathways associated with fatty acid elongation, starch and sucrose metabolism, and DNA replication. The 797 downregulated DEGs were significantly enriched in 79 pathways, including photosynthesis, glyoxylate and dicarboxylate metabolism, pentose phosphate pathway, carbon metabolism, and terpenoid backbone biosynthesis.

Overall, these transcriptional changes illustrate a bidirectional regulatory pattern, in which XOS simultaneously enhances immune responses and attenuates core metabolic functions such as photosynthesis.

### 2.6. Analysis of Biotic Stress-Related Pathways

**Figure 5:**
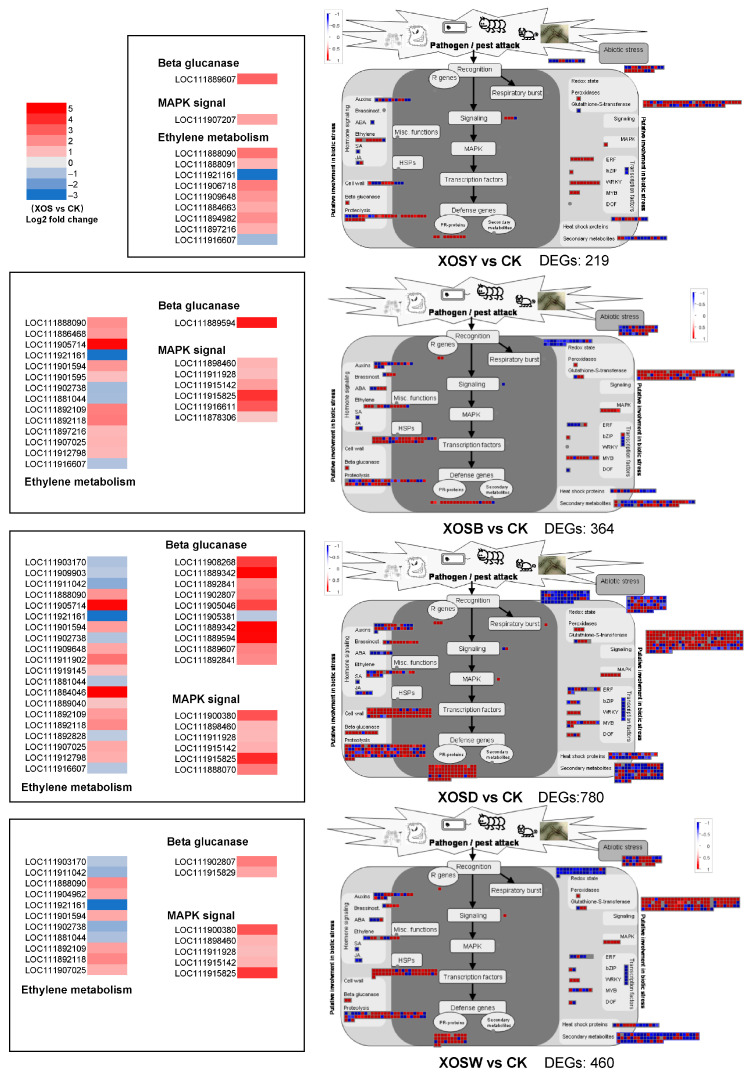
MapMan analysis of differentially expressed genes (DEGs) associated with plant biotic stress in lettuce leaves treated with xylo-oligosaccharides (DP 2–5).

To further resolve the impact of XOS on defense-associated transcriptional networks, biotic stress-related DEGs were visualized using MapMan (Figure 5). The number of DEGs associated with biotic stress varied considerably among treatments compared with the control (CK). Specifically, XOSY regulated 219 such genes, XOSB 364, XOSD 780, and XOSW 460. In the XOSD group, robust induction was observed for genes encoding β-1,3-glucanases (2–4.8-fold), MAPK kinases (six members, 1–4-fold), and pathogenesis-related (PR) proteins (66 members, 0.7–5-fold). These gene categories are central to plant immune responses and were markedly more responsive under XOSD treatment.

Hormone signaling modules also displayed DP-specific regulation. Ethylene-responsive genes showed divergent expression patterns between DP2 and DP3–5 treatments, with only two genes (LOC111888090 and LOC111921161) commonly regulated across DP3–5. Collectively, higher-DP treatments (DP3–5) engaged a larger set of hormone-regulated genes relative to DP2.

Defense transcription factor families exhibited treatment-specific signatures. XOSY upregulated seven ERF and nine WRKY genes, whereas XOSB primarily activated eight MYB and one ERF gene. XOSD produced a broader response, with four ERF, eight MYB, and four WRKY genes significantly induced. XOSW upregulated one ERF, three MYB, and three WRKY members.

In addition, genes involved in cell wall remodeling were consistently upregulated across all treatments. These included cellulose synthases and cell wall–degrading enzymes, suggesting that reinforcement and restructuring of the cell wall represent a common defense strategy under XOS exposure.

### 2.7. Analysis of Growth-Related Pathways

DEGs associated with growth-related processes varied substantially among XOS treatments (Table S3). In general, XOS exposure suppressed the expression of a subset of genes linked to carbohydrate and energy metabolism. Nevertheless, specific metabolic modules, including amino sugar and nucleotide sugar metabolism, pentose and glucuronate interconversion, pyruvate metabolism, and starch and sucrose metabolism, contained significantly upregulated genes, with starch and sucrose metabolism being most prominent.

Treatment-specific differences were evident. XOSY induced only limited changes, with a few downregulated DEGs in carbohydrate metabolism and photosynthesis pathways, and minimal upregulation. In contrast, DP3–5 (XOSB, XOSD, XOSW) upregulated DEGs across 15 carbohydrate metabolism pathways, with significant enrichment in starch and sucrose metabolism for XOSD and XOSW. At the same time, these treatments downregulated large numbers of DEGs associated with carbon fixation, nitrogen metabolism, photosynthesis, and photosystem antenna proteins, indicating a broad shift away from photosynthetic processes.

**Figure 6:**
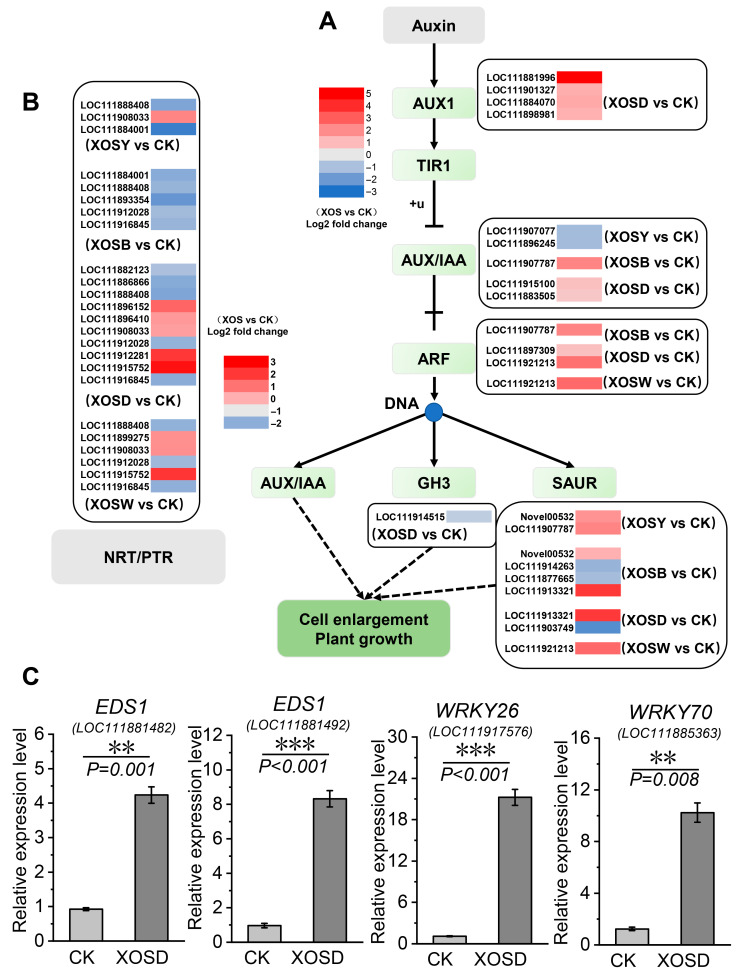
Key genes involved in the auxin signal transduction pathway (**A**), nitrate transporter proteins (**B**), and plant growth-immune balance regulation (**C**) in lettuce treated with xylo-oligosaccharides (DP 2–5). The data represent means ± SD (*n* = 3). *p*-values were derived by a two-tailed Student’s *t*-test. In the figure, ** indicates *p* < 0.01 and *** indicates *p* < 0.001, with statistically significant differences.

DEGs involved in auxin signaling and nitrate transport were strongly induced under DP4 treatment (Figure 6). Genes encoding AUX1, an auxin influx carrier (e.g., LOC111881996, LOC111901327, LOC111884070, LOC111898981), showed 1–5-fold induction. ARF transcription factors (LOC111897309, LOC111921213) were upregulated 0.9–2.6-fold (Figure 6A). DP4 treatment modulated 10 members of the NRT1 nitrate transporter family (Figure 6B). Among them, NPF5.6-like (LOC111912281) and NPF8.1-like (LOC111915752) were notably upregulated by 3- and 3.5-fold, respectively. Several nitrate transporter genes were also upregulated in the DP5 group, such as LOC111915752, LOC111899275, and LOC111998033.

Notably, growth–defense regulatory hubs were strongly induced by XOSD (Figure 6C). EDS1 genes (LOC111881482, LOC111881492) showed 3.57- and 7.63-fold induction, while WRKY transcription factors were prominently upregulated, including WRKY26 (18.51-fold) and WRKY70 (7.32-fold). Together, these transcriptional shifts highlight DP4 as the most effective treatment in simultaneously activating carbohydrate metabolism, auxin signaling, and nutrient transport, while repressing photosynthesis-related pathways.

## 3. Discussion

This study demonstrates that foliar xylo-oligosaccharides (XOS) elicit degree-of-polymerization (DP)–dependent transcriptional and phenotypic responses in lettuce. Across DP2–DP5, growth promotion and transcriptome reprogramming scaled with chain length, with xylotetraose (DP4, XOSD) consistently producing the strongest effects on shoot biomass and root architecture (Figure 1, Table S1) and the largest set of differentially expressed genes (DEGs) (Figure 2). These findings align with the principle that oligosaccharide bioactivity is conditioned by DP and molecular features [25,26].

A simple “more DP, more defense” narrative does not explain our data. Functional enrichment analyses converged on a coherent pattern: photosynthesis- and nitrogen metabolism–related genes were broadly downregulated, whereas modules linked to carbohydrate metabolism, cell wall and surface barrier formation, and immune signaling were preferentially upregulated (Figure 3 and Figure 4). Low DPs primarily repress photosynthetic gene modules with little activation of compensatory metabolism, which aligns with their limited biomass effects. In contrast, DP4–DP5 combined suppression of chlorophyll/antenna and carbon fixation pathways with induction of starch and sucrose metabolism, pentose and glucuronate interconversion, and amino sugar/nucleotide sugar metabolism (Figure 4). The signature points to metabolic reallocation from light-driven carbon assimilation toward soluble carbohydrate routes that can sustain growth under immune activation. This is a more economical way to support cell expansion when photosynthetic machinery is transcriptionally dialed down, and it explains why DP4 enhances biomass despite photosynthetic gene repression.

Integrated MapMan and GO/KEGG analyses indicate a DP-dependent expansion of defense programs. With increasing chain length, enrichment progresses from perception and early signaling to transcriptional control and downstream execution modules linked to cell-wall organization and cuticle-associated lipid pathways (Figure 3, Figure 4 and Figure 5, Tables S3 and S4). Under DP4, genes encoding β-1,3-glucanases, pathogenesis-related (PR) proteins, and MAPK cascade components are strongly induced, together with wall-associated categories, a configuration consistent with basal immune activation coupled to barrier reinforcement [27,28,29,30,31,32]. The transcription-factor repertoire also broadens with DP: DP2 is dominated by ERF and WRKY, whereas DP4 engages ERF/WRKY/MYB triads positioned to connect stress signaling to metabolic and structural outputs. The expansion of this regulatory spectrum, together with concurrent induction of defense enzymes and wall-associated terms, indicates multi-layer recruitment rather than a proportional amplification of a fixed program.

A classic concern is the growth–defense tradeoff. DP4 challenges the assumption that stronger defense invariably costs growth. We observed co-induction of AUX1 and ARF nodes together with a suite of NRT1/NPF nitrate transporters, plus upregulation of starch/sucrose metabolism (Figure 6). One plausible interpretation is that DP4 enhances sink strength and nutrient import while providing carbon substrates that buffer growth during immune activation. Concomitant induction of EDS1, WRKY26, and WRKY70 suggests a regulatory configuration that elevates defense readiness but restrains late overactivation, which aligns with frameworks where EDS1–hormone cross-talk fine-tunes costs [33,34,35,36,37,38]. The conceptual advance here is not “defense without cost” but “cost-aware defense,” in which plants rebalance energy and substrates to keep growth on track.

DP dependence is a shared motif among carbohydrate ligands, yet which DP is optimal can be species- and tissue-specific. Our data place lettuce in a regime where DP4 is optimal under our conditions, while DP5 remains active but less comprehensive. We do not claim a universal DP4 optimum across taxa. Instead, we propose that each host–ligand pair exhibits a response surface over DP and dose, with local optima that depend on receptor availability, wall context, and signaling thresholds. This perspective motivates systematic DP dose matrices in future work to distinguish potency from efficacy and to map iso-response contours.

In summary, our study establishes that XOS elicits DP-dependent transcriptional and physiological responses in lettuce, with xylotetraose (DP4) emerging as the most potent variant. DP4 simultaneously activates immune pathways and promotes growth through metabolic reprogramming, auxin signaling, and nitrate transport, highlighting a molecular basis for resolving the growth–defense tradeoff. Future research should investigate the receptor-mediated recognition of XOS with different chain lengths and explore their application as biostimulants for improving crop resilience under combined biotic and abiotic stress conditions.

## 4. Materials and Methods

### 4.1. Plant Materials

This study was conducted in a controlled-environment chamber of the Institute of Biotechnology and Food Science, Hebei Academy of Agricultural and Forestry Sciences, China, from October 2021 to April 2023. Lettuce (*Lactuca sativa var. ramosa Hort.*), cultivar ‘Roma’, was used as the experimental plant. Xylo-oligosaccharides (XOS), including xylobiose (XOSY), xylotriose (XOSB), xylotetraose (XOSD), and xylopentose (XOSW), were employed as oligosaccharide materials with a purity of 90–95%. The molecular weights of XOSY, XOSB, XOSD, and XOSW were 282.24, 414.36, 546.47, and 678.59 Da, respectively.

### 4.2. Experimental Design

Lettuce seedlings were grown hydroponically. Seeds were sown in seedling trays and germinated in darkness at 25 °C. After 80% germination, trays were transferred to a controlled chamber with an LED lighting system comprising a red (peak at 665 nm) and blue (peak at 430 nm) light combination. The light intensity was set to 240 μmol·m^−2^·s^−1^ with a 12-h photoperiod (7:00 AM–7:00 PM). The day/night temperatures were 25/20 °C, relative humidity was maintained at 50%, and the CO_2_ concentration was ambient.

When seedlings reached the two-leaf and one-heart stage (14 days old), uniform plants were selected and transplanted into light-proof hydroponic boxes (60 × 60 × 10 cm), with 9 seedlings per box. The nutrient solution (volume: 33.6 L; depth: 9.5 cm) consisted of the following components (mmol·L^−1^): 0.75 K_2_SO_4_, 0.5 KH_2_PO_4_, 0.1 KCl, 0.65 MgSO_4_·7H_2_O, 0.1 EDTA-Fe, 1.0 × 10^−3^ H_3_BO_3_, 1.0 × 10^−3^ MnSO_4_·4H_2_O, 1.0 × 10^−3^ ZnSO_4_·7H_2_O, 1.0 × 10^−4^ CuSO_4_·5H_2_O, and 5.0 × 10^−6^ H_2_MoO_4_. Calcium nitrate [Ca(NO_3_)_2_·4H_2_O] was added to provide 10 mmol·L^−1^ nitrogen. The stock nutrient solution was diluted 100-fold, and the pH was adjusted to 6.0. The nutrient solution was not replaced during the growth period.

At 16 days, seedlings were subjected to foliar application of 50 mg·L^−1^ XOS solutions (XOSY, XOSB, XOSD, or XOSW) or distilled water as the control (CK). Treatments were applied every three days, for a total of four applications, with spraying performed at 9:00 AM. The application volume was adjusted to ensure leaves were fully covered without dripping. Three hours after the final treatment, third to fifth leaves were collected, rinsed with ultrapure water, blotted dry, snap-frozen in liquid nitrogen, and stored at −80 °C for RNA extraction. Growth-related parameters were measured two days after the final treatment.

### 4.3. Measurement of Growth Parameters

Growth parameters were measured on 10 plants per treatment. The aboveground and belowground parts were separated at the root-shoot junction. Fresh weight of the shoots was measured, and total leaf area was determined using a leaf area scanner (LI-3000C, Li-Cor Biosciences, Lincoln, NE, USA). Roots were imaged using the WinRHIZO root analysis system (Regent Instruments, Quebec, QC, Canada) to obtain root morphological parameters. Dry weights of the shoots and roots were measured after drying at 70 °C for 48 h following an initial inactivation step at 105 °C for 15 min.

### 4.4. Transcriptomic Analysis

(1) RNA Extraction and Library Construction
  Total RNA was extracted using the TRIzol method (Invitrogen, Carlsbad, CA, USA) and evaluated for purity using a Nanodrop spectrophotometer (IMPLEN, Westlake Village, CA, USA) and for integrity using an Agilent 2100 Bioanalyzer (Agilent Technologies, Santa Clara, CA, USA). Samples with an RNA integrity number (RIN) greater than 7.0 were used for library construction. Ribosomal RNA was removed using the Ribo-Zero™ rRNA Removal Kit (Illumina, San Diego, CA, USA), and mRNA was enriched for sequencing. RNA samples passing quality control were sent to Beijing Novogene for library preparation. Libraries were sequenced on an Illumina NovaSeq 6000 platform using 150 bp paired-end reads.
(2) Sequencing Data Processing
  Raw reads were filtered using Trimmomatic (v0.33) to remove adapters, reads with more than 10% “N” content, and reads with low-quality bases (Q ≤ 20) exceeding 50%. Clean reads were aligned to the reference genome using STAR (v2.5.2b). Gene expression was quantified as fragments per kilobase of transcript per million mapped reads (FPKM) using HTSeq (v0.5.4.p3), and only genes with an FPKM value greater than 1 were included in subsequent analyses.
(3) Differentially Expressed Gene (DEG) Analysis
  Gene-level read counts were analyzed for differential expression using DESeq (v1.10.1). Genes with q-values < 0.05 (adjusted using the Benjamini-Hochberg method) were considered significantly differentially expressed. GO (Gene Ontology) and KEGG (Kyoto Encyclopedia of Genes and Genomes) enrichment analyses were performed for the DEGs, and MapMan was used to analyze metabolic pathways.

### 4.5. RT-qPCR Validation

Six genes were randomly selected for validation using RT-qPCR. LsPP2AA3 was used as the internal reference gene. Primer sequences are listed in Table S5. Gene expression levels were normalized to the CK, and results confirmed that RT-qPCR trends were consistent with RNA-seq data, validating the reliability of transcriptomic results.

## Supplementary Materials

The following supporting information can be downloaded at: https://www.mdpi.com/article/10.3390/plants14192958/s1, Figure S1: Comprehensive assessment of RNA-seq data accuracy; Table S1: Root morphology characteristics of lettuce plants treated with xylo-oligosaccharides (DP 2–5); Table S2: Sequencing data quality and alignment results with the reference genome; Table S3: Key metabolic pathways associated with lettuce growth under xylo-oligosaccharide (DP 2–5) treatment; Table S4: KEGG pathway enrichment of downregulated genes in lettuce leaves treated with xylooligosaccharides (DP 2–5). Table S5: Specific primers used for RT-qPCR amplification.

## Date Availability Statement

The RNA-seq data have been deposited in the Sequence Read Archive (SRA) of the National Center for Biotechnology Information (NCBI) under the accession number PRJNA1161149. All data supporting the conclusions of this study are included within the main manuscript and the supplementary files.
