# Supplementary material for: Transcriptomic Insights into the Degree of Polymerization-Dependent Bioactivity of Xylo-Oligosaccharides
Source: Plants (Basel). 2025 Sep 24;14(19):2958. doi: 10.3390/plants14192958 (PMC12525876; doi:10.3390/plants14192958)
Supplement: Supplementary file 1 [file plants-14-02958-s001.zip › plants-3777995-supplementary.pdf]

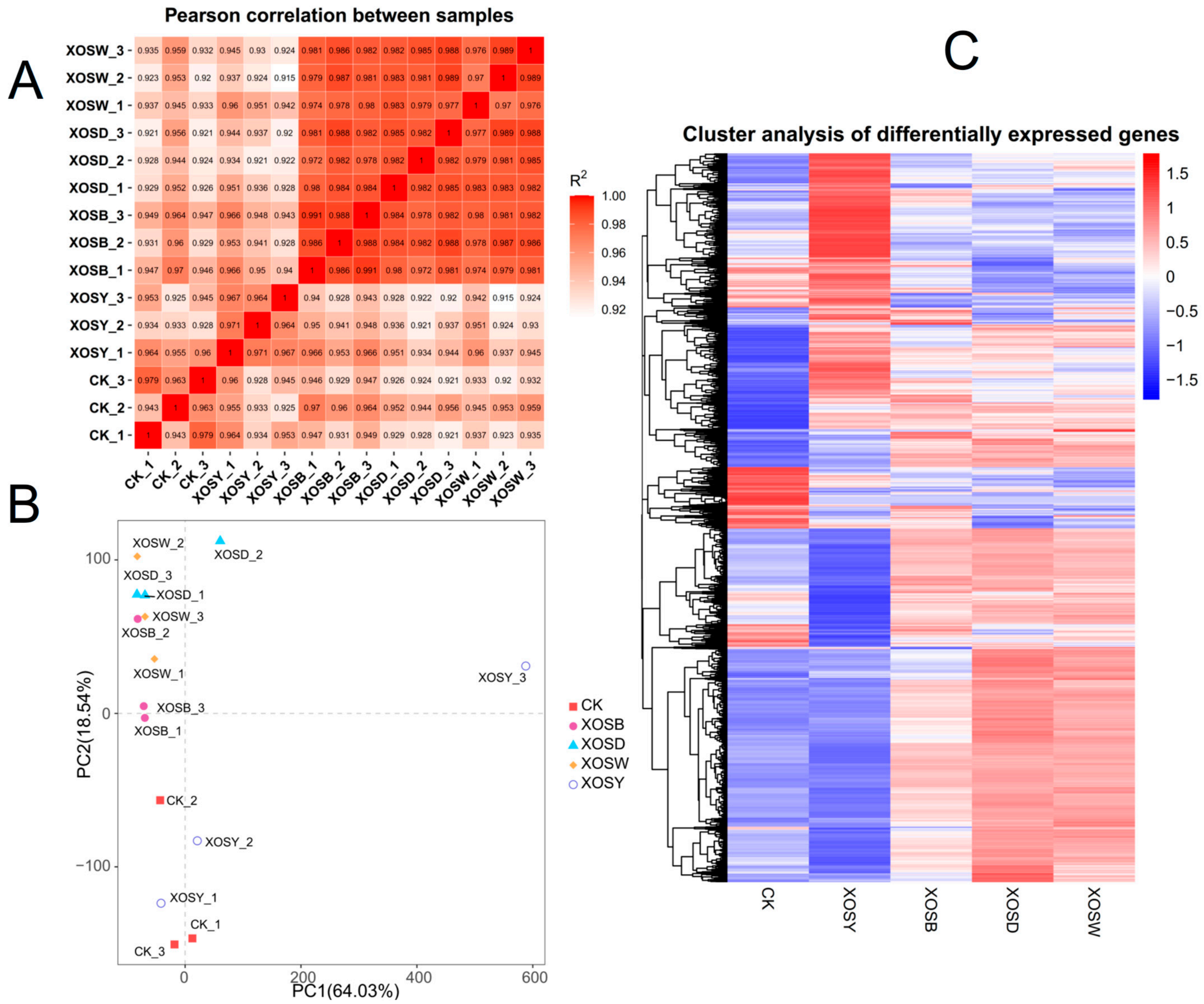

Figure S1. Comprehensive assessment of RNA-seq data accuracy. (A) Correlation analysis demonstrating the consistency and reliability of biological replicates based on pairwise Pearson correlation coefficients. (B) Principal component analysis (PCA) visualizing the variance and grouping of samples with and without xylooligosaccharides (DP 2–5) treatment. (C) Heatmap of clustered differentially expressed genes (DEGs), showcasing distinct expression patterns and clustering of samples under different treatment conditions.

Table S1. Root morphology characteristics of lettuce plants treated with xylooligosaccharides (DP 2 – 5).

Treatments	Total root length (cm)	SurfArea (cm^2)	AvgDiam (mm)	RootVolume (cm^3)	Lateral root length (cm) 0<.L.≤0.25	Adventitious root length (cm) 0.25<.L.
CK	4261.05±548.12b	303.08±42.58b	0.23±0.00ab	1.72±0.26b	1005.55±219.26b	3170.14±217.58b
XOSY	4466.73±308.35ab	318.13±15.50b	0.23±0.00b	1.87±0.10ab	1139.73±116.65b	3374.93±243.57ab
XOSB	4676.98±403.86ab	317.15±20.80ab	0.23±0.00ab	1.80±0.11ab	1091.44±67.55b	3280.46±300.10b
XOSD	5060.64±437.54a	361.12±32.62a	0.24±0.00a	2.10±0.16a	1395.92±104.47a	3838.44±350.78a
XOSW	4627.98±300.73ab	337.95±21.16ab	0.24±0.01a	1.97±0.12ab	1203.73±69.34ab	3423.84±233.09ab

Note: Means ± SD (n=6) followed by the same letter within a column are not significantly different at the 0.05 probability level according to the least significant difference (LSD) test.

Table S2. Sequencing data quality and alignment results with the reference genome.

Sample	Raw Reads	Clean Reads	Clean Base（G）	Error Rat（%）	Q30（%）	GC Content（%）
CK_1	47879030	45906234	6.89G	0.0003	0.93	0.47
CK_2	44248664	43336030	6.50G	0.0003	0.94	0.47
CK_3	46726616	45382788	6.81G	0.0003	0.94	0.47
XOSY_1	45216960	43740650	6.56G	0.0003	0.94	0.46
XOSY_2	52820254	49889950	7.48G	0.0003	0.93	0.46
XOSY_3	38907234	36385590	5.46G	0.0003	0.93	0.49
XOSB_1	45636912	44686692	6.70G	0.0003	0.93	0.46
XOSB_2	46551434	45748678	6.86G	0.0003	0.93	0.46
XOSB_3	45001846	43840756	6.58G	0.0003	0.93	0.46
XOSD_1	39036270	38125434	5.72G	0.0003	0.93	0.46
XOSD_2	55290836	53501390	8.03G	0.0003	0.94	0.47
XOSD_3	39555362	38806908	5.82G	0.0003	0.93	0.46
XOSW_1	45147652	43954154	6.59G	0.0003	0.93	0.46
XOSW_2	43784662	43122258	6.47G	0.0003	0.93	0.46
XOSW_3	41556596	40738546	6.11G	0.0003	0.94	0.46

Table S3. Key metabolic pathways associated with lettuce growth under xylooligosaccharide (DP 2 – 5) treatment.

	KEGG Terms	Up-regulated DEGs				Down-regulated DEGs			
		XOSY	XOSB	XOSD	XOSW	XOSY	XOSB	XOSD	XOSW
Carbohydrate metabolism	Amino sugar and nucleotide sugar metabolism	—	5	11	6	4	2	7	3
	Ascorbate and aldarate metabolism	—	2	5	3	—	3	10	7
	Butanoate metabolism	—	1	2	—	2	2	5	2
	C5-Branched dibasic acid metabolism	—	1	2	—	2	—	4	—
	Citrate cycle (TCA cycle)	—	2	2	2	3	4	10	3
	Fructose and mannose metabolism	—	2	3	2	5	4	11	5
	Galactose metabolism	—	1	8	6	2	2	5	3
	Glycolysis / Gluconeogenesis	—	8	9	7	8	8	23	11
	Glyoxylate and dicarboxylate metabolism	—	2	2	3	8	15	23	16
	Inositol phosphate metabolism	—	3	9	2	2	3	5	—
	Pentose and glucuronate interconversions	—	5	13	7	2	5	7	4
	Pentose phosphate pathway	—	1	—	—	6	8	16	11
	Propanoate metabolism	—	1	5	3	2	—	2	—
	Pyruvate metabolism	1	4	7	5	6	6	16	7
	Starch and sucrose metabolism	4	7	25	17	8	5	11	7
Energy metabolism	Carbon fixation in photosynthetic organisms	—	—	—	—	9	19	31	22
	Nitrogen metabolism	—	—	1	—	5	7	8	7
	Photosynthesis	—	—	—	1	1	17	37	30
	Photosynthesis-antenna proteins	—	—	—	—	2	15	22	23
	Oxidative phosphorylation	—	—	1	—	2	4	19	6

Table S4: KEGG pathway enrichment of downregulated genes in lettuce leaves treated with xylooligosaccharides (DP 2–5).

XOSY

#Term	ID	Input number	P-Value	Corrected P-Value
**Purine metabolism**	**lsv00230**	**9**	**0.00321256**	**0.091926937**
**Biosynthesis of amino acids**	**lsv01230**	**15**	**0.003649608**	**0.091926937**
**Carbon fixation in photosynthetic organisms**	**lsv00710**	**8**	**0.003655434**	**0.091926937**
**Carbon metabolism**	**lsv01200**	**16**	**0.00483826**	**0.091926937**
**Glyoxylate and dicarboxylate metabolism**	**lsv00630**	**7**	**0.009067608**	**0.137827637**
**RNA transport**	**lsv03013**	**10**	**0.018392129**	**0.215554942**
**Pentose phosphate pathway**	**lsv00030**	**5**	**0.019853745**	**0.215554942**
**Nitrogen metabolism**	**lsv00910**	**4**	**0.026492338**	**0.251677211**
**Alanine, aspartate and glutamate metabolism**	**lsv00250**	**4**	**0.043250315**	**0.365224886**
Porphyrin and chlorophyll metabolism	lsv00860	4	0.064782401	0.460114653
Vitamin B6 metabolism	lsv00750	2	0.066595542	0.460114653
Glycine, serine and threonine metabolism	lsv00260	4	0.087419364	0.534956401
Pyruvate metabolism	lsv00620	5	0.0915057	0.534956401
Selenocompound metabolism	lsv00450	2	0.102044233	0.553954407
Glycolysis / Gluconeogenesis	lsv00010	7	0.127456199	0.608456449

XOSB

#Term	ID	Input number	P-Value	Corrected P-Value
**Photosynthesis - antenna proteins**	**lsv00196**	**14**	**5.98E-11**	**4.66E-09**
**Photosynthesis**	**lsv00195**	**18**	**4.21E-09**	**1.64E-07**
**Carbon fixation in photosynthetic organisms**	**lsv00710**	**18**	**5.56E-08**	**1.45E-06**
**Carbon metabolism**	**lsv01200**	**29**	**3.75E-06**	**7.30E-05**
**Glyoxylate and dicarboxylate metabolism**	**lsv00630**	**14**	**1.17E-05**	**0.000182098**
**Porphyrin and chlorophyll metabolism**	**lsv00860**	**10**	**0.00010604**	**0.001378523**
**Nitrogen metabolism**	**lsv00910**	**6**	**0.006250472**	**0.069648117**
**Pentose phosphate pathway**	**lsv00030**	**7**	**0.007549625**	**0.073608847**
**Terpenoid backbone biosynthesis**	**lsv00900**	**8**	**0.012703721**	**0.110098917**
**Vitamin B6 metabolism**	**lsv00750**	**3**	**0.024300728**	**0.189545675**
**Biosynthesis of amino acids**	**lsv01230**	**16**	**0.034438168**	**0.229980792**
**Glycine, serine and threonine metabolism**	**lsv00260**	**6**	**0.03538166**	**0.229980792**
Biosynthesis of secondary metabolites	lsv01110	59	0.058970266	0.353821597
Thiamine metabolism	lsv00730	3	0.104300336	0.559213326
Metabolic pathways	lsv01100	107	0.12201639	0.559213326

XOSD

**#Term**	**ID**	**Input number**	**P-Value**	**Corrected P-Value**
**Photosynthesis - antenna proteins**	**lsv00196**	**24**	**1.76E-14**	**1.78E-12**
**Photosynthesis**	**lsv00195**	**34**	**2.47E-13**	**1.25E-11**
**Carbon fixation in photosynthetic organisms**	**lsv00710**	**30**	**3.23E-09**	**1.09E-07**
**Carbon metabolism**	**lsv01200**	**57**	**1.09E-08**	**2.74E-07**
**Glyoxylate and dicarboxylate metabolism**	**lsv00630**	**22**	**1.22E-05**	**0.000246922**
**Pentose phosphate pathway**	**lsv00030**	**15**	**0.000222723**	**0.003749175**
**Porphyrin and chlorophyll metabolism**	**lsv00860**	**14**	**0.000561698**	**0.008104493**
**Glycine, serine and threonine metabolism**	**lsv00260**	**13**	**0.003634936**	**0.045891069**

**Biosynthesis of amino acids**	**lsv01230**	**33**	**0.009255371**	**0.103865827**
**Glycolysis / Gluconeogenesis**	**lsv00010**	**22**	**0.015930827**	**0.160901357**
**Sulfur metabolism**	**lsv00920**	**7**	**0.022788579**	**0.209240593**
**Biosynthesis of secondary metabolites**	**lsv01110**	**125**	**0.02487915**	**0.209399509**
**Ascorbate and aldarate metabolism**	**lsv00053**	**9**	**0.04044416**	**0.30271135**
**Pyruvate metabolism**	**lsv00620**	**13**	**0.041959989**	**0.30271135**
**C5-Branched dibasic acid metabolism**	**lsv00660**	**3**	**0.047185709**	**0.317717104**

XOSW

#Term	ID	Input number	P-Value	Corrected P-Value
**Photosynthesis - antenna proteins**	**lsv00196**	**21**	**1.44E-16**	**1.14E-14**
**Photosynthesis**	**lsv00195**	**28**	**3.68E-15**	**1.45E-13**
**Carbon fixation in photosynthetic organisms**	**lsv00710**	**21**	**1.69E-08**	**4.46E-07**
**Carbon metabolism**	**lsv01200**	**37**	**1.35E-07**	**2.66E-06**
**Glyoxylate and dicarboxylate metabolism**	**lsv00630**	**17**	**2.36E-06**	**3.72E-05**
**Pentose phosphate pathway**	**lsv00030**	**10**	**0.000657134**	**0.008652265**
**Terpenoid backbone biosynthesis**	**lsv00900**	**11**	**0.002051239**	**0.020723845**
**Porphyrin and chlorophyll metabolism**	**lsv00860**	**9**	**0.002098617**	**0.020723845**
**Biosynthesis of amino acids**	**lsv01230**	**21**	**0.011039498**	**0.09690226**
**Biosynthesis of secondary metabolites**	**lsv01110**	**77**	**0.014971552**	**0.118275257**
**Nitrogen metabolism**	**lsv00910**	**6**	**0.016743831**	**0.12025115**
**Sulfur metabolism**	**lsv00920**	**5**	**0.02166608**	**0.142635027**
**Glycine, serine and threonine metabolism**	**lsv00260**	**7**	**0.033395071**	**0.202939276**
**Vitamin B6 metabolism**	**lsv00750**	**3**	**0.041928564**	**0.236596896**

Table S5. Specific primers used for RT-qPCR amplification.

ID Gene id	Gene-description	Primers sequences	RNA-seq (log2FC)	RT-qPCR (Log2FC)
LOC111904911<br>XOSY vs CK	calcium-binding protein CML38-like	F:TGGTTTCGTTTCGTCTCAGTAT<br>R:GAGTAAAATTCCTTGGTTCCAGAT	2.74	0.77
LOC111917576<br>XOSD vs CK	probable WRKY transcription factor 26	F:GGTTATAGATGGAGGAAATATGGAC<br>R:TTGTTATTGGTGTTACCGTTGTTAG	2.06	18.51
LOC111876531<br>XOSY vs CK	pathogenesis-related genes transcriptional activator PTI6-like	F:GCTGCCTCCTTTGGGTTT<br>R:GTGTCTCCTGTTTCTGCTTCG	1.05	1.15
LOC111886653<br>XOSY vs CK	pathogenesis-related genes transcriptional activator PTI6-like	F:ATCCGAATAGGAGGAAAAGAGT<br>R:TTCAATTTAACGGCAGCATC	2.03	6.22
LOC111881482<br>XOSD vs CK	protein EDS1-like	F:GGCGACTGTTTGAGCCTTTAG<br>R:GGCGACTGTTTGAGCCTTTAG	2.47	3.57
LOC111881492<br>XOSD vs CK	protein EDS1-like	F:ACATTTGAAGGGAGATGGGTAT<br>R:GTATTTTATCATCAGAACGCCAC	2.07	7.64
